# Language as a coordination tool evolves slowly

**DOI:** 10.1098/rsos.160259

**Published:** 2016-12-21

**Authors:** Tamas David-Barrett, Robin I. M. Dunbar

**Affiliations:** 1 Department of Experimental Psychology, University of Oxford, South Parks Road, Oxford OX1 3UD, UK; 2 Facultad de Gobierno, CICS, Universidad del Desarrollo, Av. Plaza 680, San Carlos de Apoquindo, Las Condes, Santiago de Chile 7610658, Chile; 3 Kiel Institute for the World Economy, Kiellinie 66, 24105 Kiel, Germany; 4 Population Research Institute, Väestöliitto, Kalevankatu 16, Helsinki 00101, Finland; 5 Department of Computer Science, Aalto University School of Science, PO Box 15500, Espoo 00076, Finland

**Keywords:** social coordination, communication efficiency, social group size, agent-based models, costs of communication, language evolution

## Abstract

Social living ultimately depends on coordination between group members, and communication is necessary to make this possible. We suggest that this might have been the key selection pressure acting on the evolution of language in humans and use a behavioural coordination model to explore the impact of communication efficiency on social group coordination. We show that when language production is expensive but there is an individual benefit to the efficiency with which individuals coordinate their behaviour, the evolution of efficient communication is selected for. Contrary to some views of language evolution, the speed of evolution is necessarily slow because there is no advantage in some individuals evolving communication abilities that much exceed those of the community at large. However, once a threshold competence has been achieved, evolution of higher order language skills may indeed be precipitate.

## 1. Introduction

Explaining the evolution of the large, complex, in some cases technologically advanced, societies of mostly unrelated individuals such as those characteristics of the more advanced mammals like primates and humans has been a challenge to biologists. All apes live in groups with relatively complex structure, and at least some level of technological culture. However, great ape communities cannot grow much beyond 40–50 individuals [1,2]. Ape sociality based on intensive, expensive, stable and long-lasting dyads is a limiting factor for the size of these societies [2,3]. By contrast, human groups are potentially limitless in size, and complexity. Maintaining coordination in such large groups requires both a large brain and the ability to communicate third-party information [4,5]. Third-party information, however, cannot be passed on without some form of language because this requires both time and place to be marked as well as the ability to identify third parties and comment on their actions.

Language of any kind has appeared only a few times in evolution, and arguably complex language evolved only once. This is a conundrum: given how useful humans find language for a host of different functions, its rarity is surprising. One possible explanation is that language might have evolved as a side effect of some other unrelated trait, and thus might be an evolutionary accident. If this explanation is true, then we would expect to see complex language emerging in a relatively short time once the simple form appears as a side effect. An alternative explanation might be that the circumstances in which complex human language evolved were unique, resulting in the phenomenon of language being unique. This explanation would allow a slower process in which the unique circumstances rather than the lucky ‘mutation’ would be driving the appearance of complex language.

While there is a problem of cheating in all forms of communication [6–9], the use of language to pass on third-party information opens up two new forms of collective action problem. First, individuals can lie about the information that they received from others [10]. This is different from primary lies, as it is more difficult to detect indirect lies of this kind. The usual solution is network-embedded reputation, rather than repeated games [11,12].

However, even if all the dishonest signalling problems are resolved, a second form of collective action problem arises. If the producing and processing of linguistic information is costly, then it might be in the interests of the individual to freeride on others' (costly) ability to produce efficient coordination. This condition is clearly present in humans. The production of human language requires an apparatus that is particularly costly: it required a set of changes to human breathing that increases mortality risk, substantially increased cognitive processing power and, hence, an energetically more costly brain [13,14]. Why has the production of this public good not collapsed via less good linguists freeriding on the collective benefits provided by others while saving on their individual costs?

One possible reason is that human language might be functionally tuned for mate choice purposes [15], or directly useful for other reasons. While we do not doubt that, once it has evolved, language provides a capacity that can be used in many different contexts where it might be exploited by, for example, sexual selection, the fact that language's capacity to pass on third-party information has evolved extremely rarely does suggest that these features are secondary [16,17]. Instead, we argue that the primary origin of language lies in coordinating relatively large-scale collective action [4], that language exists to enable cooperation on a large scale, either as an ecological signalling system [18] or a means of social coordination [19,20]. For a group to perform a complex task, its members need to coordinate their actions using a pre-agreed set of signs and signals.

To investigate this problem, we use an agent-based behavioural synchrony model [21] with two novel distinguishing characteristics. First, we use a primate-like two-level social network in which each individual interacts only with a small subset of its social group [22–24]. Second, we use the group's behavioural synchrony as a necessary condition for group action in what is, in effect, a two-step process: in order to solve an important ecological problem it is first necessary to create a cohesive social group of some specified size because it is the group that allows the problem to be solved [25]. In other words, it is behavioural coordination that makes social action (such as defence against predators or coordinated foraging routes) possible. The fitness payoff to the individual members of a community depends both on the size of the group and on how well synchronized their behaviour is with each other [26]. Although we cast our model in terms of language, we argue that it applies more generally to all communication systems whenever communication is targeted at facilitating coordination and/or social cohesion through coordinated or synchronized action.

We use compass direction as a simple exemplar of synchronized action, but it represents any kind of behavioural synchrony (e.g. agreeing on a social rule or a cultural icon) that ensures collective action. In this model, each member of a group of agents is assigned a random vector on a dial (i.e. a compass direction). This is their initial information about the social action of the group. Agents go through a set of one-on-one meetings with those to whom they are socially linked. At each meeting, they exchange information about their respective vectors. However, each agent receives this information with noise (a trait of the recipient rather than the sender of the information). Using this new (but noisy) information, the agents reset their own vector values midway on the dial between their own previous value and the (noisy) one they received from the agent they have just met. This set-up allows us to take the noise element to be an inverse proxy for the efficiency of communication (in effect, the efficiency of language): the more precise the information transfer during an encounter, the more effective the process of synchronization will be. Improving the effectiveness of communication by reducing the error term then represents the costs of evolving language (or, indeed, any other communication system).

Once synchronization is achieved, the group performs the communal action. The better they manage to coordinate, the more the group gets out of this action (and the higher the individual fitness of the ingroup members). As information transmission noise slows down the rate of coordination (or convergence), the higher the average noise level the further the agents end up from each other, and thus the lower the benefit from the communal action. Put more explicitly, if coordination efficiency declines the less effectively the group evades predators, the less successful it is in foraging. While the benefit each individual receives from the group action is the same for everyone, each agent's payoff is modified by two costs: her vector value's distance from everyone else's at the end of the synchronization process, and the cost she is willing to invest to reduce the noise she faces when receiving information. Thus, this model poses a classic public goods problem: the low noise level is a public good in that it creates a benefit for all (faster, better synchronization), which can only be achieved via the individuals' costly contributions (reducing noise). The problem of linguistic coordination thus adds a solution to the public goods problem via a cost that the agents face from poor individual-level coordination, which is intrinsic to coordination and thus does not require third-party policing. This way language that, all else equal, should pose a public goods conundrum simply bypasses the problem if it emerges as a coordination tool.

## 2. Methods

First, we build a model of linguistic coordination of collective action in a group.

We assume that the agents form a network such that each agent is connected to the same number of others as every other group member. Thus to introduce a group of agents mathematically, let *G*(*n*) denote the set of all connected *k*-regular *n*-sized graphs

2.1 $$G(n)=\{g(n)|\#\;e_{i}=k\;\forall \;i,\rho (i,j)<\infty \;\forall \;i,j\},$$

where *e_i_* denotes the set of agents that agent *i* is connected to and *ρ*(*i*,*j*) denotes the network distance between nodes *i* and *j*.

Note that the definition in equation (2.1) implies that all the elements of the *G* set are connected graphs. We use connected graphs, as we are interested in how a group of agents that form one social network can coordinate collective action. If they are not connected, then the problem becomes trivial as no group-level coordination becomes possible. In other words, we assume that the agents form a social network that can be defined as a group.

Our definition of the set *G* also implies that we are only interested in *k*-regular graphs, i.e. networks in which each agent is connected to the same number of others. This is based on the observation that if maintaining relationships is costly then the social network is unlikely to be fully connected [21], a claim that is supported by the fact that the variation of meaningful social relationships is limited in both non-human primates, as well as in humans [27,28].

We assume that the agents are coordinating a collective action problem, represented in the model by coordination on a unit circle. Thus, at time *t* = 0, each agent starts with a random vector on a dial, a standard representation of group synchrony problem

2.2 $$\phi_{0,i}∼U(0^{\circ },360^{\circ })\;\forall \;i,$$

where $\phi_{0,i}$ are independently and identically distributed.

When the agents receive new information, an agent updates her direction to midway between her current direction and the perceived direction of the interactant

2.3 $$f(\phi_{i},\phi_{j})=\left\{ \begin{array}{ll} f_{1} & \text{if}\;0^{\circ }\leq f_{1}\leq 360^{\circ }\\ f_{1}-360^{\circ } & \text{if}\;360^{\circ }<f_{1}\\ f_{1}+360^{\circ } & \text{if}\;f_{1}\leq 0^{\circ }, \end{array}\right.$$

where

2.4 $$f_{1}(\phi_{i},\phi_{j})=\left\{ \begin{array}{ll} \frac{\phi_{i}\;+\;\phi_{j}}{2} & \text{if}\;0^{\circ }\leq |\phi_{i}-\phi_{j}|\leq 180^{\circ }\\ \frac{\phi_{i}\;+\;\phi_{j}\;+\;360^{\circ }}{2} & \text{if}\;180^{\circ }<\phi_{i}-\phi_{j}\\ \frac{\phi_{i}\;+\;\phi_{j}\;-\;360^{\circ }}{2} & \text{if}\;\phi_{i}-\phi_{j}<-180^{\circ }, \end{array}\right.$$

where *ϕ_i_* and *ϕ_j_* are information vectors of agents *i* and *j*.

We place the coordination problem on a unit circle rather than on a linear range, as in the former no single exchange of information can provide any guidance as to where the group's coordination converges, and hence the agents cannot shortcut the coordination process.

A synchronization event on the graph *g*(*n*)∈*G*(*n*) is a series of *T* meetings among the agents, where a ‘meeting’ between two agents, *i* and *j*, is an interaction between connected agents, with each pair of connected agents equally likely to meet

2.5 $$\{i,j\}∼U{\left\{1,2,\ldots ,n\right\}}^{2}\;\text{s.t.}\;i\neq j,i\in e_{j},j\in e_{i},$$

that is, the agents meet only agents that they themselves are connected to.

So far the set-up is the same as in [4]. However, in the present model, we assume that the agents' communication includes an error factor on the side of the information recipient. Thus, when agent *i* receives information from a partner she perceives it perturbed by a noise characteristic to her, $\varepsilon_{i}$. In other words, each agent is coupled with a noise variable that is specific to their reception of the information.

Thus, in a meeting between agents *i* and *j*, after exchanging their information, both agents update their respective information the following way

2.6 $$\begin{array}{rl} \phi_{t+1,a} & =f(\phi_{t,a},\phi_{t,b}+\nu_{a})\\ \;\text{and}\;\phi_{t+1,b} & =f(\phi_{t,b},\phi_{t,a}+\nu_{b}), \end{array}\}$$

where $\nu_{a}∼\{-\varepsilon_{a},\varepsilon_{a}\}$ is a random variable that is either a positive or negative deviation from the information value.

Meetings are repeated until *t* = *T*, where *T* = *τn*/2, i.e. until on average each agent participates in *τ* information exchanges.

Let *d_i_* denote the individual *i*'s average distance from the others at *T*, the endpoint of the synchronization event

2.7 $$d_{i,j}=\frac{1}{n-1}\sum_{j\neq i}^{n}\left|\phi_{T,i}-\phi_{T,j}\right|,$$

where we applied the same convention as in (2.4).

Let $\bar{d\;}\;$ denote the average distance among the agents

2.8 $$\bar{d\;}\;=\frac{1}{n}\sum_{j=1}^{n}d_{j}.$$

We assume that the group's payoff from the collective action is dependent on the efficiency of coordination in a way that the less efficient the coordination, the lower the group-level payoff. Thus, let *C* denote the group-level payoff, and let *H* denote the net group payoff the following way:

2.9 $$H=C-D\bar{d\;}\;,$$

where *D* is the parameter of losing payoff due to inefficiency of coordination. We assume that *D* > 0.

Let us assume that the agent's individual payoff is made up of the following elements: her share from the group's payoff reduced by her distance to the other agents, and by the cost of reducing her noise level. Thus her payoff, *H_i_*, is determined the following way:

2.10 $$H_{i}=\frac{1}{n}H-Ad_{i}-B(180-\varepsilon_{i}),$$

where *A* is the cost parameter associated with the individual's information being distant to other individuals, and *B* is the cost of reducing the noise of the incoming information.

Combining equations (2.9) and (2.10)

2.11 $$H_{i}=\frac{1}{n}C-\frac{1}{n}D\bar{d\;}\;-Ad_{i}-B(180-\varepsilon_{i}).$$

As we assumed above that *D* > 0, we can normalize the payoff function the following way:

2.12 $$\frac{H_{i}n}{D}=\frac{C}{D}-\bar{d\;}\;-\frac{nA}{D}d_{i}-\frac{nB}{D}(180-\varepsilon_{i}),$$

thus

2.13 $$h_{i}=-\bar{d\;}\;-\alpha d_{i}-\beta (180-\varepsilon_{i}),$$

where $h_{i}=(nH_{i}-C)\text{/}D$, α=nA/D and β=nB/D. The parameter *α* can be interpreted as the agent's individual cost of being inefficient at updating the information, and the parameter *β* can be interpreted as the cost of communication precision.

Note that as *C* and *D* are fixed parameters and the group size, *n* is fixed, the agent *i*'s maximization of the payoff *h_i_* in $\varepsilon_{i}$ is equivalent to the optimum in *H_i_*. Hence, it is possible to examine the individual's optimization problem simplified to

2.14 $$\underset{\varepsilon_{i}}{\max}h_{i}{|}_{\alpha ,\beta }.$$

In other words, the optimization problem involves choosing the right noise level, given both the cost of being inefficient at coordination and the cost of increasing communication precision.

Note that this set-up implies two important characteristics of this coordination problem. First, the agents are naive in the primary coordination problem: they communicate information to others in a non-strategic way. Second, however, there is a secondary element, the noise level, which is the proxy for linguistic ability, in which there is space for agents to alter their individual payoffs. Assuming that *β* is positive (which is equivalent to the assumption that both *B* and *D* parameters are positive), then if *α* is small enough implies that there would be a conflict of interest between the group and the noise level. In this case, the model simplifies to the classic tragedy of commons case. The inclusion of the second term in (2.13) thus allows a solution to the standard free rider problem in a way that the cost term comes as an intrinsic consequence of coordination, and not as third-party policing.

## 3. Results

**Figure 1.**
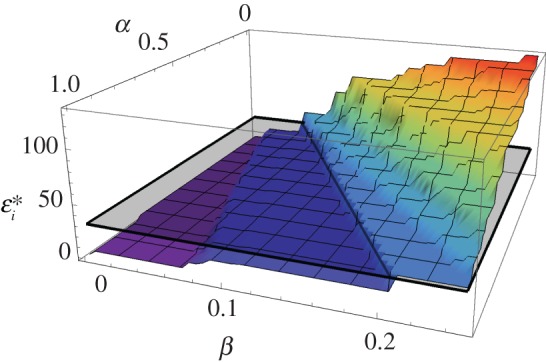
The effect of the cost parameters on the optimal noise level of an individual agent: in some parameter range, it is optimal for the agent to have less efficient language than the others, but elsewhere an improvement is optimal. The letters *α* and *β* denote the cost parameters associated, respectively, with the degree of vector synchrony (average distance on the dial from others) and precision (inverse noise). The symbol $\varepsilon_{i}^{*}$ denotes the optimal noise level of agent *i*. The noise level of all other agents is fixed, and represented by the grey pane. (Average of 100 repeats, *n* = 10, *k* = 4, *τ* = 40; the noise level of all others is fixed at 30.)

To examine the incentives for the individual, let us fix the noise level of all agents except for the focal agent *i* (figure 1). As expected, the results show that the optimal noise level is dependent on both parameters *α* (the agent's individual cost of being inefficient at updating the information) and *β* (the general cost of communication precision). More importantly, however, the optimal noise level is above that of everyone else in the group for some parameters, while smaller than that of the others in some other parameter ranges. Note that in the former case the agent can be seen as freeriding on others, while in the latter case the agent is a net contributor to the collective action.

Note that the tendency towards freeriding is moderated by a cost of being far from the others at the end of the synchronization process. In effect, this creates a trade-off. If the cost of *communication precision* is relatively high, it is beneficial for the agent to freeride on others; but if the cost of *being individually inefficient at coordination* is relatively high, then it is beneficial for the agent to tolerate a lower noise level than others.

Although, mathematically, this structure is trivial, when applied to language it raises some interesting questions. Although it has been claimed that linguistic information is strategic, to our knowledge it has not been shown that the ability to communicate in complex language is also open to free riders, and thus could easily result in a tragedy of commons. Perhaps, the reason behind the rarity of complex language lies exactly in this point: it might just be likely that *β* tends to be above *α* in almost all natural settings.

Assuming that communication precision, i.e. the variable set ${\left\{\varepsilon_{i}\right\}}_{i=1}^{n}$, is inherited by agents, we introduce an evolutionary mechanism based on the payoffs. To do this, we assume a simple evolutionary dynamics such that, at each evolutionary step, the noise level of the agent with the smallest payoff is replaced by that of the highest payoff, thereby mimicking differential selection favouring the more successful individuals: in each step, the agent *i* with the lowest payoff is eliminated, and replaced with a new agent, still labelled *i*. The new agent *i*'s value of $\varepsilon_{i}$ is chosen using that of the highest-payoff agent from the previous round (say, *j*), with an error

3.1 $${\left\{\varepsilon_{s+,i}\right\}}_{i=1}^{n}={\left\{\varepsilon_{s,i}\right\}}_{i=1}^{n}\backslash \varepsilon_{s,\mathrm{drop}}\cup \varepsilon_{s,\mathrm{new}}|h_{s,\mathrm{drop}}=\min{\left\{h_{s,i}\right\}}_{i=1}^{n}$$

where

3.2 $$\varepsilon_{s,\mathrm{new}}∼U((1-\theta )\varepsilon_{s,\mathrm{top}},(1+\theta )\varepsilon_{s,\mathrm{top}})|h_{s,\mathrm{top}}=\max{\left\{h_{s,i}\right\}}_{i=1}^{n},$$

where *s* is an evolutionary time step, and *θ* is the parameter of copying error. (Note that the evolutionary mechanism in equations (3.1) and (3.2) assumes that the payoff from the collective action, captured in parameter *C*, is high enough so that all payoffs are positive, i.e. *h_i_* > 0 for all *i*. We return to this assumption below.)

**Figure 2.**
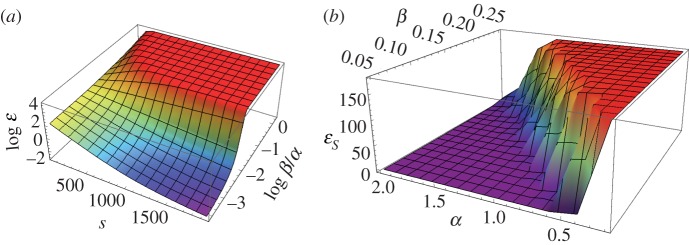
Efficient language evolves if the individual's cost of producing it is under a threshold level. (*a*) The evolution of noise level (communication efficiency) as a function of the ratio of the cost parameters. *s* denotes evolutionary time; *α* and *β* denote the cost parameters (figure 1); and *ϵ* is the average noise level of the group. (*b*) Average noise level at the end of the evolutionary process as a function of the two cost parameters. The symbol $\varepsilon_{s}$ denotes average noise level of the group at the end of the evolutionary process. (Average of 40 repeats, *n* = 10, *k* = 4, *τ* = 40, $\varepsilon_{0}=10$, the variable ε ranges between 0 and 180.)

Not surprisingly, the ratio of the cost parameters *α* and *β* determines the way the group's noise level evolves. If the cost of precision is high, then freeriding is evolutionarily advantageous, and noise levels quickly converge to maximum (figure 2*a*). If this cost is less forbidding, then the group ends up with a low level of noise in communication for every member of the group, and thus a more efficient coordination of their communal action. The final evolutionary state is entirely dependent on the ratio of the two costs with a rapid phase transition between the two states (figure 2*b*).

Thus, in some parameter range the classic collapse of public goods emerges: high communication costs trigger freeriding among the agents, leading to the collapse of coordination efficiency, and a collapse in the group's ability to perform the collective action that the coordination is designed to achieve. However, elsewhere within the parameter range, all agents choose to make costly investments towards a shared communication efficiency, as the opportunity cost incurred by losing efficiency in the precision of their individual coordination decisions is even more costly.

The evolution of low-noise communication lends itself to being interpreted in terms of language evolution. Language is a communication tool that facilitates the coordination of group action: language is costly for the individual to produce, but failure to invest in linguistic ability results in reduced benefits from group action.

This suggests that language could have evolved as a tool for coordinating collective action within a group providing the following conditions hold:

(a) The cost of being an inefficient coordinator individually is relatively high compared with the cost of increasing communication precision. That is, the agent's lost share of the group payoff from collective action due to their inability to coordinate well is larger than the cost of producing efficient language.
(b) The payoff from the group's collective action is high enough to exceed the cost of the agent's communication precision at all points during the evolutionary process. Even if the cost of communication efficiency is smaller than the payoff at the very end of the evolution of language, the process would not get going if it is high at the very beginning. Hence, this account of language evolution is as much about the underlying collective action as about the individual costs.

This implies that the size of the group plays a role. To examine whether this is so, first let us ignore the fact that the payoff has to be positive (so that the agents actually survive). In other words, we assume that the parameter *C* in equation (2.9) is always high enough so that all payoffs are positive. However, let us assume that for this payoff to materialize, that is, for the group to survive, the average distance among the agents has to be below a threshold

3.3 $$\bar{d\;}\;\leq \lambda ,$$

where λ is an arbitrary parameter. This is equivalent to changing equation (2.9) to the following:

3.4 $$H=c-D\bar{d\;}\;,$$

where

3.5 $$c=\left\{ \begin{array}{ll} C & \text{if}\;\bar{d\;}\;\leq \lambda \\ 0 & \text{if}\;\bar{d\;}\;>\lambda , \end{array}\right.$$

and we assume that the group can only survive if *H* > 0.

The rationale behind introducing the limit, λ, lies with the assumption that groups that are unable to get to a minimum level of efficiency in coordinating their collective action get nothing out of trying to do so. To see why this is important, note that the average distance among the agents at the end of a synchronization event, $\bar{d\;}\;$, increases with group size, i.e. $\partial \bar{d\;}\;/\partial n>0$, a property established elsewhere [4]. Thus, it is meaningful to ask how large a group can be and still be able to perform a collective action given the limit of λ in (3.3).

Let *n*_max_ denote the maximum size of group that can achieve behavioural synchrony given a particular error level. That is,

3.6 $$n_{\max}=\max n|\bar{d\;}\;\leq \lambda .$$

**Figure 3.**
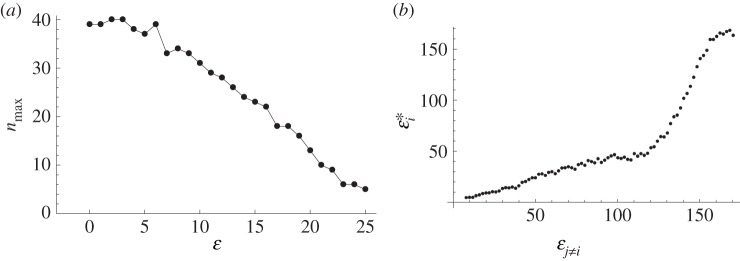
The evolution of language allows larger groups to emerge, but is a relatively slow process. (*a*) Falling noise level facilitates the emergence of larger groups. (*b*) When the optimal noise level of the individual, $\varepsilon_{i}^{*}$, is dependent on the average noise level of the others in the group, language emerges slowly. In effect, it is suboptimal to have to have a much better language capacity than the rest of the group. The cost parameter ratio was set at 0.1, ensuring that there is a convergence to the low noise. (Average of 100 repeats, *α* = 1, *β* = 0.1, *n* = 10, *k* = 4, *τ* = 40.)

The results suggest that, as the noise level falls, maximum group size increases (figure 3*a*). In other words, the evolution of more precise communication allows larger group sizes, in line with the role that language is thought to have played in human evolution [20,29]. Many primate and human groups can draw a benefit from coordinating a larger group, for instance, from a disproportionately increased efficiency in exploiting the environment, or from maintaining higher technological complexity, or from defence against predators or neighbouring conspecific groups. If that is the case, the increased coordination efficiency allowed by the fall of noise in our model, i.e. the rise of complex language in our interpretation, would lead to increasing group size.

Note that in the two alternative paths of evolution (figure 2*a*) there is a remarkable difference in the speed of reaching the long-term steady state: while the collapse is fast, the evolution of low noise levels is slow. The precision of communication thus emerges relatively slowly at first (figure 3*b*): even if it is optimal for the individual to improve precision, it is suboptimal to be much better than the rest of the group. In other words, being a relatively good communicator might provide individual benefits, driving the slow evolution of language forward. However, if the individual is much better in communication ability than the rest of the group then the advantage is limited, while the cost is high. As far as the efficiency of group coordination is concerned, there is no benefit in being a significantly more efficient communicator than other members of your community. This makes intuitive sense: if a hypothetical group of modern day humans would be learning a new language together, there would be no point for any one individual to jump ahead to being a fluent speaker while everyone else in the group were still beginners. If such a jump is very costly, which would have been the case with the evolution of linguistic abilities, then it would be outright disadvantageous to move much faster than the rest of the group. This suggests that if language evolved primarily as a coordination tool, its evolution was a slow process that required a pressure towards larger groups with more efficient coordination for a protracted period of evolutionary time.

## 4. Discussion

We have presented a model of behavioural synchrony in which we have shown that the ability to communicate with language can be perceived as a free rider problem when the primary purpose of language use is the coordination of the group. We have shown it is possible to portray the ‘punishment term’ of the collective action problem as intrinsic to coordination, and hence the presence of a third-party punisher is not needed. This may explain how language could have emerged despite the fact that it seems to pose a classic free rider problem.

Humans are unique among animals in their propensity to cooperate with non-kin in large groups [30]. Hitherto, research on this topic has been concerned mainly with how the collective action paradox is resolved either spontaneously [31], via peer [32–34] or pool [35,36] punishment, via reputational mechanisms [11,12], or via costly communication signals [37,38]. A problem, however, has been that it is not clear why or how the human ability to form the groups that produce public goods from the costly contributions of the individuals evolved in the first place [39].

As a possible solution to this problem, we introduced a model of collective action defined in terms of synchrony [4,21,40] which uses behavioural coordination [41] on structured social networks [42]. In our model, all agents are naive, and the possible incentive incompatibility in the dyadic interactions is solved by the stability of the dyads that are maintained via frequently repeated interactions. Instead, we observe that even if the objective of the individual was to cooperate, there is a crucial assumption lying behind the cooperation being linguistic: the ability to produce and process language.

Our model shows that if the essence of cooperation is to achieve behavioural synchrony using a language-like signalling process, then the collective action paradox disappears as long as small improvements in the individual's language ability are not forbiddingly expensive. This is in line with recent findings on the effect of group synchrony on human physiology [43,44], and suggests that the evolution of language, sensitivity towards physical synchrony and the propensity for cooperation could well have emerged in a coevolutionary process.

Why is language so rare, when it is so useful? Our model suggests that the answer may be that it has to provide an evolutionary benefit right at the outset of the emergence of communication, and continue to do so all the way through the evolutionary process. This seemingly trivial point translates into the observation that for complex language to emerge, there must be an ecological advantage of increased group size that outweighs the cost of further precision of communication. This suggests that human language must have evolved stepwise with progressively increasing complexity. This is in line with evidence from primates that both gestural and vocal communication channels respond in just this way to increasing group size. Among anthropoid primate species, the size and complexity of the vocal repertoire correlates with social group size [45], and the same appears to be true of gestural communication [46], although this may be subject to a lower asymptotic limit [25]. Similarly, among chickadees (a North American passerine) vocal complexity increases facultatively with social group size in both the wild and captivity [47].

The results allow us to draw four important novel conclusions, all of which alter the conventional wisdom on language evolution. First, our model relies on language as a mechanism for coordinating behaviour, not for the exchange of factual information: doing so allows language to evolve explicitly as a social function in a way that builds on the natural functions of gestural and vocal communication in primates. This does not mean that language cannot be used for the exchange of information, but it does suggest that this was a secondary function.

Second, at the same time, this approach circumvents the collective action paradox in the sense that a ‘punishment term’, which in conventional models implies a third-party punisher, can—in the case of language—be perceived as a naturally intrinsic phenomenon of language.

Third, the acquisition of efficient language facilitates the evolution of progressively larger social groups (which becomes critical when community size determines the benefits of sociality). The fact that language explicitly allows agents to coordinate larger groups has not previously been considered, either in terms of language evolution or for the evolution of cooperation.

Fourth, the process of language evolution is, at least initially, slow and cumulative, requiring language communities to evolve increasing competences stepwise. Hitherto, the consensus has been that language must have evolved rapidly [48]. Our results suggest that this is not necessarily so; indeed, our model suggests that evolving capacities that are significantly better than those of the rest of the community may actually be disadvantageous.

These results have important implications for how we view both human sociality and the evolution of cooperation in the human context, both of which have been of considerable interest to the social and evolutionary sciences. The conventional view of human sociality, in particular, is that members of a group exchange resources or information of some kind for which they pay a cost, with dyadic exchanges being the typical model for all such interactions. In such cases, they must recover that cost either by an equivalent trade (I give you apples in exchange for your oranges) or by the reciprocation of the benefit over time (I help you out now, but I expect you to help me out in return at some point in the near future). This is essentially an economist's view of sociality (cooperation as a trading relationship), and we suggest that it has been the main cause of the collective action paradox which has bedevilled attempts to explain the evolution of large-scale cooperation. By contrast, our model is based on the view that, in the anthropoid primate and human cases (and perhaps those of a number of other intensely social taxa), cooperative solutions to key ecological problems are an emergent property of behavioural synchrony at group level [4,49]. In primates, the principal selection factor favouring group-living is protection against predators [50–52], and continues to be so in humans [53], where predators are to be understood as being either conventional carnivores or conspecific raiders [54–56]. However, solving that ecological problem requires that the individual animals can form and maintain a coordinated, cohesive social group.

In such cases, the effectiveness of defence is provided by the size of the group, and the size of the group is simply a function of how well individuals can coordinate their behaviour so as to remain together. The cost that is paid for doing so is minimal, because individuals are not explicitly involved in active defence—it is the simple presence of the group that deters predators and raiders, not the actions of individual group members [51,57]. More importantly, they are not engaged in exchanging any kind of commodity (including information), and therefore individual animals cannot face the prospect that others might contribute less than they do. As a result, the free rider problem that gives rise to the collective action paradox is not so intrusive: an individual cannot ‘pay’ more or less than anyone else for the benefit gained from defence—it simply joins the club or it does not, and it benefits accordingly (and may pay a heavier cost if it doesn't join by incurring a higher predation risk if it forages alone or in a smaller group [51,57]). Taken together, these results imply that human sociality is less about dyadic exchange and more about achieving social coordination at the community level.

## Data accessibility

All results are contained by the model, no data was used.

## References

[1] Campbell CJ 2007 Primates in perspective, xiv, 720 p. New York, NY: Oxford University Press.

[2] Lehmann J, Korstjens AH, Dunbar RTM 2008 Time and distribution: a model of ape biogeography. Ethol. Ecol. Evol. 20, 337–359. (doi:10.1080/08927014.2008.9522516)

[3] Lehmann J, Korstjens AH, Dunbar RIM 2007 Group size, grooming and social cohesion in primates. Anim. Behav. 74, 1617–1629. (doi:10.1016/j.anbehav.2006.10.025)

[4] David-Barrett T, Dunbar RIM 2013 Processing power limits social group size: computational evidence for the cognitive costs of sociality. Proc R. Soc. B 280, 20131151 (doi:10.1098/rspb.2013.1151) 10.1098/rspb.2013.1151 PMC3712454 23804623

[5] Dunbar RI, Shultz S 2007 Evolution in the social brain. Science 317, 1344–1347. (doi:10.1126/science.1145463) 17823343 10.1126/science.1145463

[6] Lachmann M, Szamado S, Bergstrom CT 2001 Cost and conflict in animal signals and human language. Proc. Natl Acad. Sci. USA 98, 13 189–13 194. (doi:10.1073/pnas.231216498) 10.1073/pnas.231216498 PMC60846 11687618

[7] Lachmann M, Bergstrom CT 1998 Signalling among relatives—II. Beyond the tower of Babel. Theor. Popul. Biol. 54, 146–160. (doi:10.1006/tpbi.1997.1372) 9733656 10.1006/tpbi.1997.1372

[8] Bergstrom CT, Lachmann M 1998 Signaling among relatives. III. Talk is cheap. Proc. Natl Acad. Sci. USA 95, 5100–5105. (doi:10.1073/pnas.95.9.5100) 9560235 10.1073/pnas.95.9.5100 PMC20220

[9] Bergstrom CT, Lachmann M 1997 Signalling among relatives .1. Is costly signalling too costly? Phil. Trans R. Soc Lond. B 352, 609–617. (doi:10.1098/rstb.1997.0041)

[10] Iniguez G, Govezensky T, Dunbar R, Kaski K, Barrio RA 2014 Effects of deception in social networks. Proc. R. Soc. B 281, 20141195 (doi:10.1098/rspb.2014.1195) 10.1098/rspb.2014.1195 PMC4123708 25056625

[11] Nowak MA, Sigmund K 2005 Evolution of indirect reciprocity. Nature 437, 1291–1298. (doi:10.1038/nature04131) 16251955 10.1038/nature04131

[12] Wedekind C, Milinski M 2000 Cooperation through image scoring in humans. Science 288, 850–852. (doi:10.1126/science.288.5467.850) 10797005 10.1126/science.288.5467.850

[13] Aiello LC, Wheeler P 1995 The expensive-tissue hypothesis—the brain and the digestive-system in human and primate evolution. Curr. Anthropol. 36, 199–221. (doi:10.1086/204350)

[14] Isler K, van Schaik CP 2006 Metabolic costs of brain size evolution. Biol. Lett. 2, 557–560. (doi:10.1098/rsbl.2006.0538) 17148287 10.1098/rsbl.2006.0538 PMC1834002

[15] Miller G 1999 Sexual selection for cultural displays. In The evolution of culture (eds Dunbar RIM, Knight C, Power C), pp. 71–91. Edinburgh, UK: Edinburgh University Press.

[16] Dunbar RIM 2009 Why only humans have language. In The prehistory of language (eds Botha R, Knight C), pp. 12–35. Oxford, UK: Oxford University Press.

[17] Redhead G, Dunbar RIM 2013 The functions of language: an experimental study. Evol. Psychol. 11, 845–854. 23945312 10.1177/147470491301100409 PMC10481022

[18] Tomasello M 2008 Origins of human communication, xiii, 393 p. Cambridge, MA: MIT.

[19] Deacon TW 1997 The symbolic species: the co-evolution of language and the human brain, 527 pp. London, UK: Penguin.

[20] Dunbar RIM 1996 Grooming, gossip and the evolution of language, 230 p. London, UK: Faber.

[21] David-Barrett T, Dunbar RI 2012 Cooperation, behavioural synchrony and status in social networks. J. Theor. Biol. 308, 88–95. (doi:10.1016/j.jtbi.2012.05.007) 22609470 10.1016/j.jtbi.2012.05.007

[22] Zhou WX, Sornette D, Hill RA, Dunbar RIM 2005 Discrete hierarchical organization of social group sizes. Proc. R. Soc. B 272, 439–444. (doi:10.1098/rspb.2004.2970) 10.1098/rspb.2004.2970 PMC1634986 15734699

[23] Hill RA, Bentley RA, Dunbar RIM 2008 Network scaling reveals consistent fractal pattern in hierarchical mammalian societies. Biol. Lett. 4, 748–751. (doi:10.1098/rsbl.2008.0393) 18765349 10.1098/rsbl.2008.0393 PMC2614163

[24] Dávid-Barrett T, Carney J 2015 The deification of historical figures and the emergence of priesthoods as a solution to a network coordination problem. Religion Brain Behav. 2015, 1–11.

[25] Dunbar RIM 2012 Bridging the bonding gap: the transition from primates to humans. Phil. Trans. R. Soc. B 367, 1837–1846. (doi:10.1098/rstb.2011.0217) 22641822 10.1098/rstb.2011.0217 PMC3367699

[26] Conradt L, List C 2009 Group decisions in humans and animals: a survey. Phil. Trans. R. Soc. B 364, 719–742. (doi:10.1098/rstb.2008.0276) 19073475 10.1098/rstb.2008.0276 PMC2689721

[27] Sutcliffe A, Dunbar R, Binder J, Arrow H 2012 Relationships and the social brain: Integrating psychological and evolutionary perspectives. Br. J. Psychol. 103, 149–168. (doi:10.1111/j.2044-8295.2011.02061.x) 22506741 10.1111/j.2044-8295.2011.02061.x

[28] Saramaki J, Leicht EA, Lopez E, Roberts SG, Reed-Tsochas F, Dunbar RI 2014 Persistence of social signatures in human communication. Proc. Natl Acad. Sci. USA 111, 942–947. (doi:10.1073/pnas.1308540110) 24395777 10.1073/pnas.1308540110 PMC3903242

[29] Dunbar R 1993 Coevolution of neocortex size, group size and language in humans. Behav. Brain Sci. 16, 681–735. (doi:10.1017/S0140525X00032325)

[30] Clutton-Brock T 2009 Cooperation between non-kin in animal societies. Nature 462, 51–57. (doi:10.1038/nature08366) 19890322 10.1038/nature08366

[31] Ostrom E 1990 Governing the commons: the evolution of institutions for collective action, xviii, 280 p. Cambridge, UK: Cambridge University Press.

[32] Fehr E, Gachter S 2000 Cooperation and punishment in public goods experiments. Am. Econ. Rev. 90, 980–994. (doi:10.1257/aer.90.4.980)

[33] Rockenbach B, Milinski M 2006 The efficient interaction of indirect reciprocity and costly punishment. Nature 444, 718–723. (doi:10.1038/nature05229) 17151660 10.1038/nature05229

[34] Henrich J et al. 2006 Costly punishment across human societies. Science 312, 1767–1770. (doi:10.1126/science.1127333) 16794075 10.1126/science.1127333

[35] Sigmund K, De Silva H, Traulsen A, Hauert C 2010 Social learning promotes institutions for governing the commons. Nature 466, 861–863. (doi:10.1038/nature09203) 20631710 10.1038/nature09203

[36] Boyd R, Gintis H, Bowles S 2010 Coordinated punishment of defectors sustains cooperation and can proliferate when rare. Science 328, 617–620. (doi:10.1126/science.1183665) 20431013 10.1126/science.1183665

[37] Skyrms B 2009 Evolution of signalling systems with multiple senders and receivers. Phil. Trans. R. Soc. B 364, 771–779. (doi:10.1098/rstb.2008.0258) 19073482 10.1098/rstb.2008.0258 PMC2689717

[38] Zollman KJS, Bergstrom CT, Huttegger SM 2013 Between cheap and costly signals: the evolution of partially honest communication. Proc. R. Soc. B 280, 20121878 (doi:10.1098/rspb.2012.1878) 10.1098/rspb.2012.1878 PMC3574420 23135681

[39] Burton-Chellew MN, West SA 2013 Prosocial preferences do not explain human cooperation in public-goods games. Proc. Natl Acad. Sci. USA 110, 216–221. (doi:10.1073/pnas.1210960110) 23248298 10.1073/pnas.1210960110 PMC3538240

[40] David-Barrett T, Dunbar RIM 2014 Social elites can emerge naturally when interaction in networks is restricted. Behav. Ecol. 25, 58–68. (doi:10.1093/beheco/art085)

[41] Couzin ID, Krause J, Franks NR, Levin SA 2005 Effective leadership and decision-making in animal groups on the move. Nature 433, 513–516. (doi:10.1038/nature03236) 15690039 10.1038/nature03236

[42] Young HP 2011 The dynamics of social innovation. Proc. Natl Acad. Sci. USA 108, 21 285–21 291. (doi:10.1073/pnas.1100973108) 10.1073/pnas.1100973108 PMC3271568 22198762

[43] Dunbar RIM et al. 2012 Social laughter is correlated with an elevated pain threshold. Proc. R. Soc. B 279, 1161–1167. (doi:10.1098/rspb.2011.1373) 10.1098/rspb.2011.1373 PMC3267132 21920973

[44] Bahrami B, Olsen K, Latham PE, Roepstorff A, Rees G, Frith CD 2010 Optimally interacting minds. Science 329, 1081–1085. (doi:10.1126/science.1185718) 20798320 10.1126/science.1185718 PMC3371582

[45] McComb K, Semple S 2005 Coevolution of vocal communication and sociality in primates. Biol. Lett. 1, 381–385. (doi:10.1098/rsbl.2005.0366) 17148212 10.1098/rsbl.2005.0366 PMC1626386

[46] Dobson SD 2009 Socioecological correlates of facial mobility in nonhuman anthropoids. Am. J. Phys. Anthropol. 139, 413–420. (doi:10.1002/ajpa.21007) 19235791 10.1002/ajpa.21007

[47] Freeberg TM 2006 Social complexity can drive vocal complexity: group size influences vocal information in Carolina chickadees. Psychol. Sci. 17, 557–561. (doi:10.1111/j.1467-9280.2006.01743.x) 16866738 10.1111/j.1467-9280.2006.01743.x

[48] Nowak MA, Krakauer DC 1999 The evolution of language. Proc. Natl Acad. Sci. USA 96, 8028–8033. (doi:10.1073/pnas.96.14.8028) 10393942 10.1073/pnas.96.14.8028 PMC22182

[49] Dunbar RIM, Shultz S 2010 Bondedness and sociality. Behaviour 147, 775–803. (doi:10.1163/000579510X501151)

[50] Vanschaik CP 1983 Why are diurnal primates living in groups. Behaviour 87, 120–144. (doi:10.1163/156853983X00147)

[51] Shultz S, Noe R, McGraw WS, Dunbar RIM 2004 A community-level evaluation of the impact of prey behavioural and ecological characteristics on predator diet composition. Proc. R. Soc Lond. B 271, 725–732. (doi:10.1098/rspb.2003.2626) 10.1098/rspb.2003.2626 PMC1691645 15209106

[52] Shultz S, Dunbar RIM 2007 The evolution of the social brain: anthropoid primates contrast with other vertebrates. Proc. R. Soc. B 274, 2429–2436. (doi:10.1098/rspb.2007.0693) 10.1098/rspb.2007.0693 PMC2274976 17652066

[53] Lehmann J, Lee PC, Dunbar RIM 2014 Unravelling the evolutionary function of communities. In Lucy to language: benchmark papers (eds Dunbar RIM, Gamble CS, Gowlett JAJ), pp. 245–276. Oxford, UK: Oxford University Press.

[54] Wrangham RW, Peterson D 1996 Demonic males: apes and the origins of human violence, 350 p. Boston, MA: Houghton Mifflin Company.

[55] Wrangham RW, Wilson ML, Muller MN 2006 Comparative rates of violence in chimpanzees and humans. Primates 47, 14–26. (doi:10.1007/s10329-005-0140-1) 16132168 10.1007/s10329-005-0140-1

[56] Bowles S 2009 Did warfare among ancestral hunter-gatherers affect the evolution of human social behaviors? Science 324, 1293–1298. (doi:10.1126/science.1168112) 19498163 10.1126/science.1168112

[57] Shultz S, Finlayson LV 2010 Large body and small brain and group sizes are associated with predator preferences for mammalian prey. Behav. Ecol. 21, 1073–1079. (doi:10.1093/beheco/arq108)

